# Why do students struggle in their first year of medical school? A qualitative study of student voices

**DOI:** 10.1186/s12909-022-03158-4

**Published:** 2022-02-16

**Authors:** Aled Picton, Sheila Greenfield, Jayne Parry

**Affiliations:** Institute of Applied Health Research, College of Medical and Dental Sciences, University of Birmingham, Edgbaston, Birmingham, B15 2TT UK

**Keywords:** Medical education, Undergraduate, Attrition, Struggling, Professional identity, Mental health, Support

## Abstract

**Background:**

Struggling at medical school incorporates academic failure, course disruption and early course exit. Struggling is usually multi-factorial involving academic, personal, financial and health factors. Struggling students may fail to engage with available support. First year students are particularly susceptible as they transition to university and a professional career.

**Methods:**

The study aim was to explore medical students’ own voices on struggling and assess how they match up to existing literature. During one academic year, all first year medical students at the University of Birmingham (UK) who opted to leave or were required to withdraw (*n* = 52) were asked to participate in an individual exit interview. Fifteen students responded and fourteen (27%) agreed to be interviewed. Interviews were face to face (*n* = 10), telephone (*n* = 3) and via email (*n* = 1). Interviews were unstructured and led by a general open question. Framework analysis identified key data themes.

**Results:**

Students described year one of medical school as a critical transition. They simultaneously needed to adapt to being a university student, a medical student and a doctor. A six-group typology of students emerged, each of which struggled with one or more of these adaptations. The groups were: wrong degree choice, mental health problems, acute crisis, at capacity, slow starter and family rock. Some students experienced an isolated problem from within this typology. Most had a multi-factorial story of struggling. Mental health problems and acute crises were the most common issues. Early professional identity formation was a key hurdle. Help-seeking behaviours were varied.

**Conclusions:**

This study explores the narratives of medical students who struggled from an early stage and presents a data-driven typology of their issues. It advances existing qualitative understanding of this topic, which to date is predominantly derived from educator perceptions and not specific to early course issues. Although our results broadly cohere with existing knowledge, we also present novel findings which may reflect our focus on first year students. Issues around early professional identity formation may reflect the increasing emphasis on professionalism in medical school curricula. Listening to these narratives could help university staff to identify students at risk of struggling for targeted support.

**Supplementary Information:**

The online version contains supplementary material available at 10.1186/s12909-022-03158-4.

## Introduction

10–15% of UK medical students struggle at some point in their undergraduate studies, defined as experiencing academic failure, course disruption or early course exit [1]. This may be an underestimate as some strugglers do not come to educators’ attention [2]. Alternative terms for strugglers include problem learners, at-risk students and students in difficulty [3, 4].

Many strugglers go on to leave medical courses [1]. Medical course attrition rates are relatively low compared to other university programmes and averaged at 11% in a meta-analysis of international data [5]. Single institution retrospective studies in UK medical schools have previously identified attrition rates of 6% (University of Nottingham) [6] and 14% (University of Leeds) [7]. Although the numbers of strugglers and medical course dropouts are low, reasons for struggling should be examined closely. Admission to medical school is a high-stakes decision for the student, their family, the university and society with significant investment required from all parties. There are substantial costs involved with struggling. These can be psychological, for example the affected individual and their wider family may experience distress or stigma [8]. There are also significant financial costs for the student, their family, the university and society [6].

Reasons for struggling are not always clear cut and are usually a mixture of academic, personal, social, financial and health factors [6, 9, 10]. New or pre-existing mental health problems such as anxiety, depression and eating disorders are frequently present and are recognised to either be a cause or consequence of struggling, or both [1].

Studies of psychological wellbeing in medical students indicate that at the outset of the course, their mental health is comparable to the general population [11, 12]. However, longitudinal studies identify that mental health often deteriorates through medical school. Mental health problems such as depression, anxiety and suicidal thoughts become increasingly prevalent and this trend can persist into postgraduate training [13–16]. For example, studies using validated questionnaires of psychiatric morbidity such as the General Health Questionnaire (GHQ-12) in UK medical schools identify scores indicating psychiatric ‘caseness’ in 25–37% of students [1]. These rates are higher than age-matched peers or the general population [1, 17–19]. These findings appear to corroborate the hypothesis that course-specific challenges, culture and environment at medical school can precipitate or worsen mental health issues.

Pastoral and support mechanisms exist within medical schools but may rely on students maintaining insight into their struggles and seeking help. Students who struggle may fail to engage with these supportive mechanisms, often due to concerns about confidentiality and perceived long-term effects on their career [8].

Struggling can occur at any time but first year students are particularly susceptible as they adapt to new methods of learning at university [10]. Medical students face additional, course-specific challenges. Medical cohorts are typically large and consist of academic high-flyers. This can lead to a competitive culture and contribute to the professional identity formation process, which involves students gradually learning to play the role of a doctor: ‘to pretend until they become’ [20]. Medical students’ new identity is typically based around professional inclusivity and social exclusivity [1, 21]. Professional inclusivity involves medical students feeling ‘part of the profession’ via an apprenticeship model. Social exclusivity involves medical students perceiving themselves as physically and socially separate from students in other disciplines. Professional identity formation is a key transitionary hurdle but can be particularly challenging for those that struggle if their characteristics or circumstances are different to those of their peers [22, 23].

In 2011 Hays, Lawson and Gray proposed a broad classification framework of struggling medical students. The authors developed case studies of struggling students based on real presentations to student support services. These underwent two stages of revision with approximately sixty international academic staff contributing [24]. Following this, seven profiles of strugglers were put forward, termed as follows: *immaturity, poor organisational skills, poor insight, poor mental health, major personal crisis, poor learning skills 1 and poor learning skills 2.* A student matching the poor learning skills 1 profile would be *‘more likely to be a school leaver, often strong academic performance, motivation variable, challenged by not being in top academic rank at medical school’.* In contrast the poor learning skills 2 profile is described as ‘*widening participation access, variable age and education achievement, performance poor, may have specific education defects/gaps’ *[24].

The authors advocated further empirical research to assess whether their model based on educator perceptions would hold true when compared to first-hand student narratives. In this study we respond to this need by presenting medical students’ own voices and stories of why they struggled. From these we offer a typology of strugglers and compare this to the Hays, Lawson and Gray model as well as other research findings [24].

We have focussed exclusively on the first year of study. One half of all medical course dropouts occur in the first year and it is hypothesised that this is due to different reasons than dropouts in later years [25]. Narratives from first year students may provide new insight into early course challenges.

## Methods

### Study setting

The study took place at the University of Birmingham Medical School, UK. All methods were carried out in accordance with institution guidelines and regulations. The study period is not specified to protect participant confidentiality but took place within the past 5 years.

All participants had been first year students on the 5-year Medicine and Surgery (MBChB) programme in the preceding academic year. Similar to most medicine programmes in the UK, the first year of the programme is substantially university-based with a focus on biomedical and social sciences [26]. Students spend approximately one day per month in a general practice (family-based medicine) which includes contact with patients. All students have access to pastoral and academic support including regular meetings with a Personal Academic Tutor (member of university staff or clinician) and support staff at the student services centre.

### Inclusion and exclusion criteria

Students eligible for study inclusion were identified via the end of year Examination Board as those who had:

- Opted to leave the course before taking their end of year assessments
- Opted to leave the course after their end of year assessments but before the exam board convened
- Been required to withdraw from the course based on their end of year assessment results. These participants included students who appealed this result and at the time of the study were resitting their first year, and those who did not appeal and left the course.

### Exclusion criteria

Students in Years 2–5.

### Recruitment

The intake for Year One of the MBChB programme is approximately ~ 360 students per year. Approximately two thirds of these students are female. Students who met the above inclusion criteria (*n* = 52) were contacted via email and post by JP. Students were asked to participate in an individual interview to explore their experience of the first year. Two reminders were sent to all students who did not respond. These were sent two and three weeks after the initial contact. If no response was received after the second reminder, the student was considered as not consenting to participate in the study. Of the 15 who did respond, one declined to participate in the study and the remainder (*n* = 14) proceeded to interviews.

### Consent

All students who agreed to be interviewed provided written informed consent to participate in the study.

### Interview format

Interviews (*n* = 14) took place approximately six months after the end of the academic year in question. All interviews were conducted by JP, a Professor of Policy and Public Health. Interviews were face to face (*n* = 10) or via telephone (*n* = 3). Face to face interviews were recorded on university premises. Interview length for face to face and telephone interviews ranged from 30–90 min. One student agreed to participate by answering questions via email only.

The initial part of each interview was deliberately unstructured in order to allow the interviewee to ‘tell their story’. Direct questions were avoided and only introduced later in the interview if a key point of interest identified pre-interview by the researchers had not been volunteered or addressed. These were:

- Their reasons to study medicine and to come to the University of Birmingham
- Their experience of their first academic year with key ‘events’ (e.g. realising falling behind in studies, unhappy with choice of course or university, changes in family or other relationships) mapped chronologically
- Their understanding of the availability of, and use of, sources of support
- Their relationship with staff and peers
- The advice they would now give to a new Year 1 student

Interviewees were encouraged to reflect on their responses and to offer their own interpretations of their narrative. The Interview Prompt Sheet was informed by the literature and is provided in Appendix 1.

### Data management

Interviews were transcribed ad verbatim via a commercial transcribing service. Transcripts were not returned to participants for comment or correction. Each participant and their transcript were assigned a study number [0011-0024]. Data were managed and analysed using NVivo qualitative data analysis software (QSR International Pty Ltd. Version 12, 2018).

### Coding framework

Data were analysed using framework analysis [27]. All fourteen transcripts were initially read and reread in full by the research team. The team consisted of JP, SG a Professor of Sociology and AP an academic paediatric trainee. A coding framework was developed via an iterative, data-led process. This is provided in Appendix 2. Both JP and AP developed their own coding framework based on independent coding of *n* = 2 transcripts which were selected via a random number generator. JP and AP kept coding diaries during this process.

The research team then reviewed the two coding frameworks for concordance and created a synthesised coding framework (V1). This underwent two further revisions after application to the data to form a final coding framework (V3). All interview transcripts (*n* = 14) were then coded to the V3 framework by AP to ensure coverage of themes which emerged later in the analysis. Participants did not provide feedback on the data analysis.

### Typology development

A framework coding matrix was developed which outlined each participant’s interview data against themes and subthemes of the coding framework. This provided a visual overview of clustering of each student’s data. As an example, some students’ issues were predominantly academic, evidenced by heavy coverage of the ‘studying’ theme. Analysis of this data led to development of a novel six-group typology of struggling students, based on spread of data across particular areas [28]. Each student’s narrative and spread of data were analysed in order to assign their place in the six-group typology. The initial allocation was carried out by AP. The assigned types for each student were discussed and revised with the research team to ensure agreement. We used primary and secondary issues for some students to reflect multi-factorial stories of struggling. Primary issues were deemed the most important issue to a student’s story of struggling. Some students were assigned two primary issues if the research team felt both factors made equal contributions to their narrative.

### Reflexivity statement

During the academic year in question, all researchers had occasional teaching commitments on the MBChB programme but were not core teaching faculty. They may have come into contact with the participants prior to study recruitment, but if so this would have most likely been for a single teaching session. None had a specific educational relationship (e.g. Personal Academic Tutor) with the participants. Only JP met or spoke to the study participants; AP and SG examined pseudo-anonymised transcripts only.

Both JP and AP studied at UK medical schools so in broad terms belong to the same ‘tribe’ as the participants. Belonging to this group may provide some benefits to data analysis via a shared understanding of medical school and the professional expectations placed on medical students and doctors. However social realities do not stand still and are subject to continuous change [29]. Study participants belong to Generation Z, born between 1996–2012, and as such are a different generation to the researchers [30].

As qualitative researchers, we act as ‘human instruments’ when interacting with study data [31]. There is a risk of projection from ourselves onto the data, based on our own lived experiences and biases. We have sought to reduce the risk of researcher projection through triangulation and dependability. Examples of this process include reviewing transcripts individually, comparing coding frameworks and discussing the analysis to achieve inter-researcher agreement.

### Data saturation

Data saturation was reached as enough information was achieved to replicate the study, there was no further new information to be attained from the data and further coding was not feasible [32]. The data triangulation process described above supported our conclusion that we had reached data saturation [32].

## Results

### Study participants

Fourteen students (*n* = 11 female) participated. Of these, one had completed a previous degree course (0016) and one took a gap year (0024). The remaining students had come directly from school to university. This is broadly representative of intakes to the University of Birmingham five-year degree programme, where the majority of students will be direct school leavers.

### The transition period

**Fig. 1:**
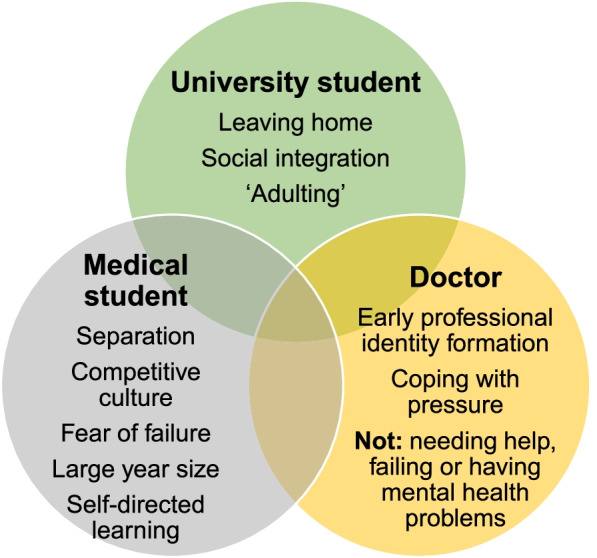
Three transitions required to succeed in year one of medical school

Eleven key themes and seventy-three sub-themes were identified in the data. Although minority viewpoints were present, analysis of these themes identified a common story: year one of medical school represented a critical transition. To succeed, a student would need to make three key adaptations which would start from day one. These adaptations involved simultaneously learning how to be a university student, a medical student and a doctor. These identities interacted and overlapped with each other, particularly those of medical student and doctor, but each presented specific challenges. The three transitions are summarised in Fig. 1.

#### University student

Several students acknowledged a struggle with independent life as a university student which could be *‘overwhelming’* (0015). Struggling with this transition contributed to poor social integration for some students:

*‘For most people…they’ve moved away from home probably for the first time, and trying to make new friends and everything like that, I’d say it’s probably quite stressful in general because it’s just out of their comfort zone.’* (0021)

University life also required a degree of ‘adulting’, defined by the Cambridge Dictionary as ‘actions or behaviours that are considered typical of adults, not children or young people’ [33]. Students frequently referenced keeping on top of daily activities such as cooking, washing and shopping. This presented an additional challenge to students’ ability to manage their time:

*‘I knew it would be quite difficult…but it was more like getting to grips with having to cook for myself every day, just the basic things as well as the work.’* (0012)

#### Medical student

Students reported course-specific challenges including separation from other university students, a competitive culture amongst high-achieving students, and fear of failure. Students reported physical separation from other students on campus:

*‘You could go to medical school for the entire five years without needing to go down to university except for exams.’* (0019)

There was also chronological separation from other students due to the number of timetabled teaching hours. This could lead to tensions with students on other courses:

*‘My flatmates couldn’t understand why I wasn’t happy for them to be up at 04:00 making lots of noise.’* (0017)

Some students voiced regret about how these separations impacted their integration into the university:

*‘A good thing about university is you’re mixing with people that are doing different courses and that think differently to you…[separation] kind of prevents that interaction between different people.’* (0017)

The educational culture amongst the cohort was at times described as stressful and competitive. Students joined a large cohort, all of whom were academic high achievers:

*‘[At school] it’s not hard to be top of the class, it’s not hard to be top of 30 people. Whereas in university, trying to be the top of 300 of the most elite students from across the country, that’s on a completely different level.’* (0024)

Participants described an increase in workload compared to their previous studies and the transition to greater self-directed learning. These were a frequent source of anxiety:

*‘A Levels to this degree: literally I can’t even explain in words how big the jump is.’* (0020)

*‘You need to know kind of all of it and you don’t know where to stop or how much you actually need to know.’* (0011)

Within this environment, fear of failure was common. All but one participant acknowledged that they had not struggled academically before, leading to a tendency to catastrophise when failure occurred:

*‘When you don’t pass something for the first time- the end of the world is here.’* (0014)

Fear of failure was usually non-specific or abstract; failure itself was unexpected and often came as a shock to students:

*‘I just feel like it was a horrific shock for us all: no-one in that group expected, like even the ones that had resits were all very confident that…we were going to get through.’* (0024)

Some students described feelings of shame about their failure:

*‘You feel like you’ve let everyone down and it was just absolutely awful.’ (0011)*

For students who were required to re-sit a year of study their examination failure was compounded by exclusion from their original cohort:

*‘At the beginning when you have that* [welcome year group] *photo and they were like, it’ll be much smaller by fifth year, you never really think like you’re the person that’s not going to be in it.’* (0012)

*‘You’re called somebody who’s resitting, you’re different, you’re not with your friends, you’ve failed.’* (0014)

#### Doctor

Participants self-identified as ‘medics’, a separate tribe within the wider population of university students. The Cambridge Dictionary defines medic as ‘a medical student or doctor’ [34]. Repeated use of this term suggests that students primarily identified as belonging to the medical profession rather than the university:

*‘I wouldn’t say that you’re part of the university…you’re very much part of a different group.’* (0017)

*‘We introduce ourselves as saying ‘Hi, I’m a medic’ rather than ‘Hi, I do medicine’…my flatmate said ‘I don’t introduce myself as a mathematician- you say ‘I do maths’…Medics love to emphasise the fact that they’re medics.’* (0011)

‘Being a medic’ was characterised as being able to manage a large workload and deal with pressure. Needing help, failing or admitting to experiencing stress or anxiety were viewed by most participants as incompatible with this identity:

*‘You’re expected to be able to cope- you’re doing medicine, you should be smart, you should be able to cope with pressure.’* (0017)

One student was admitted via a widening participation route and lived at home with their family. For this student, this created an additional layer to the professional identity formation process and marked them as different to their peers:

*‘You go home and you’ve got this home life…you don’t fit in anywhere’ (0020)*

*‘I’ve got friends who come from private schools, their whole family are doctors…their siblings are studying abroad and I was like “do I belong here?”* (0020)

### Typology of struggling students

Within the common chronology of simultaneously learning to be a university student, medical student and doctor, we were able to identify a six-group typology of struggling students [28]. Each of these groups struggled with one or more of these adaptations. We termed these groups wrong degree choice, mental health problems, acute crisis, at capacity, slow starter and family rock. The terms at capacity, slow starter and family rock are novel and are outlined in more detail below.

A minority of students (*n* = 4) experienced an isolated issue from within this typology. However, most had a multi-factorial story of struggling with both primary and secondary issues. The most frequently occurring issues were acute crises and mental health problems. The participants and their typology issues are summarised in Table 1.

**Table 1:** Summary of participants and their issues

Participant ID	Summary	Primary issue(s)	Secondary issue(s)
0011	Struggled with volume of workload. Suffered a family bereavement and subsequent low mood	At capacity	Acute crisis Mental health problems
0012	Struggled with transition to university, particularly self-directed learning and independent living	At capacity Slow starter	
0013	Struggled with workload and transition. Supported their parent through a health scare and experienced a family bereavement	At capacity Acute crisis	Family rock
0014	Physical health problems requiring inpatient care plus depression/anxiety	Acute crisis Mental health problems	
0015	Difficulties with workload, self-directed learning and time management. Struggled to settle and make friends	At capacity	Slow starter
0016	Acute stressors: family bereavement and conflict, relationship breakdown. Developed depression	Acute crisis Mental health problems	
0017	Never wanted to study medicine and didn’t enjoy the course. Developed depression and anxiety. Left to study another subject at a different university	Wrong degree choice	Mental health problems
0018	Joined course at short notice. Pre-existing mental health problems. Experienced a relationship breakdown, family conflict and significant family responsibilities	Slow starter Mental health problems	Family rock Acute crisis
0019	Suffered a bereavement which derailed studies. No issues on course prior to this	Acute crisis	
0020	Lived at home with family responsibilities during a period of bereavements and conflict. Experienced mental health problems	Family rock Acute crisis	Mental health problems
0021	Well supported with medical school application and applied as not sure what else to do. Enjoyed some aspects of course and performed well before deciding to change to a different course	Wrong degree choice	
**0022** **Email transcript**	Parental mental health issues and stress at home. Spent significant amount of time at home and fell behind on studies. Developed anxiety	Family rock	Mental health problems
0023	Initially performed well academically before falling behind due to volume of work. Changed to a different course but open-minded about applying to study medicine again in the future	At capacity	
0024	Family responsibilities, poor parental health. Spent significant amount of time at home or travelling there, fell behind on studies	Family rock	

#### Wrong degree choice

Two students provided a narrative that was distinct from the other participants and described reservations about applying for medicine:

*‘It was one of those things that I thought I could do and was capable of, but didn’t necessarily want to do it.’* (0017)

‘*I was applying because I didn’t know what else to do and it seemed like a good idea.’* (0021)

Despite these pre-existing concerns, they were well-supported with medical school applications and started the programme. One was affected by mental health problems and both quickly identified that they had chosen the wrong course:

*‘I started realising that I didn’t really like talking to patients.’* (0021)

These two students opted to leave the course at different points in the year and both went on to join different degree programmes.

### Mental health problems

These were experienced by half of students. Mental health problems were either a primary (*n* = 3 students) or secondary (*n* = 4 students) issue, but always occurred alongside other issues rather than in isolation. Anxiety and/or depression were most commonly reported, with some participants admitting their mental health problems pre-existed their admission to medical school.

Students described a pervading rumour that the medical school perceived mental health problems as incompatible with a medical career. Evidence to support the rumour was vague, for example the mention of a previous student of unknown identity having been ‘*kicked out for depression’* (0014). These beliefs were used by participants to justify non-disclosure and non-engagement with support:

*‘What if it* [acknowledging a mental health problem] *affects my medical career later?’* (0014)

*‘You’re the ones that are supposed to be treating these problems, not the ones with them.’ (0017)*

### Acute crisis

Half of the students reported a crisis which had affected their studies. Examples included physical or mental health problems requiring in-patient care, death of a family member or partner, parental divorce and family conflict. Students who experienced an acute crisis struggled with both the academic and social demands of being a medical student. Themes of strength and weakness as part of professional identity formation re-emerged when students justified not seeking support:

*‘I should be able to get through it myself, I shouldn’t need any help.’* (0019)

*‘I’m a strong person.’ (*0011*).*

Two students experienced an acute crisis due to personal health issues. In contrast to the prevailing narrative of the interviews, these students felt able to see their struggle in context and to move on:

*‘I’m not a failed medic…I was really ill.’* (0014 - physical and mental health issues)

*‘I’m fine with it because it’s a learning curve and I was ill so I put it down to that.’* (0016 - mental health issues)

### At capacity

These students’ (*n* = 5) main issues were academic: they struggled to become a medical student and meet the demands of the course. They overlapped with slow starters, mental health problems and family rocks.

Students described struggling with the transition to higher education. Pre-university schooling had emphasised outcome-based learning in a supported, teacher-led environment with a well-defined syllabus. Studying topics at university with less clearly defined end-points was described as *‘daunting’* and ‘*piecing together a puzzle with no light’* compared to school when *‘you know what you need to learn’ (0013)*. Workload and volume were major stressors:

*‘I found the workload very, very intense from the start.’* (0015)

*‘Once it got to the middle of the year I went over everything- I just don’t think that I could hold all the information.’* (0023)

At capacity students acknowledged that they struggled to adapt to self-directed learning, for example:

*‘I didn’t know how to revise.’ (0012)*

*‘I like to have things taught to me rather than doing it myself.’ (0015)*

### Slow starter

These students (*n* = 3) initially struggled with the transition to independent living at university. They reported falling behind early in the course and never catching up due to a combination of academic and social factors.

*‘I did find it quite difficult to settle in at first…I found it difficult making friends as well.’* (0015)

Once the course started, they then went on to struggle with time management and self-directed learning, for example:

*‘Because some of it’s [learning] independent, you can fall behind and not realise it.’* (0015)

Once students established that they had fallen behind compared to their peers, this was perceived as an irreversible situation:

*‘The feeling that I was behind never really went away.’ (0018).*

Students repeatedly emphasised that *‘staying on top’* (0012) would have prevented their academic struggle, but this was poorly-defined as a concept:

*‘No one really tells you how to stay on top, so you just have in the back of your mind ‘stay on top’ but without any real guidance.’ (0012)*

### Family rock

Some students had significant family responsibilities (*n* = 5). These students largely described a multi-factorial experience of struggling and overlapped with the acute crisis, slow starter and at capacity students. Mental health problems were common in both individual students and their family members.

Family rock students described playing a central role in family dynamics, often inverting traditional parent–child roles and instead being the chief source of support for both parents and siblings. For example:

*‘I assumed a huge responsibility to my family: in terms of being the base in which they cling in order to remain upright.’ (0022- email transcript).*

Some students supported family members with long-term mental health problems or disabilities:

*‘My [parent] is disabled and I do find a lot of the time I’m just around the flat, it’s a little bit hard to study sometimes because [they] need my help quite a bit.’* (0018)

Others were required in acute crises such as family bereavements or conflicts.

Students generally had insight that their family commitments had a direct impact on their academic progress and social integration at university:

‘*I found it really difficult because I’m trying to support my family, do my degree, find new friends, find my foot in this university.’* (0020)

Despite the effect on their studies, students were generally proud of their family responsibilities but acknowledged that they came at a cost:

*‘I wanted to be superwoman for my family.’ (0024).*

Of these five students, only one lived at home close to the university. The other four students with family elsewhere in the country reported travelling home every single weekend to support their family, leaving them little time to study:

‘*I tried to go home every week/fortnight to support and be with my family, which meant I had no time for work.’* (0022- email transcript)

## Discussion

First year students are particularly vulnerable to struggling during their adaptation to university life. This can lead to course failure, voluntary withdrawal, financial costs and feelings of low self-worth. For medical students these transitionary challenges are compounded by the demands of their course and the need to start to acquire a professional role.

We have proposed a data-driven typology of struggling first year medical students. Interview data from those with first-hand experience of struggling provides an additional perspective to existing literature, which to date has been largely derived from educator perceptions.

We present several new findings which emerged from these narratives. Our exclusive focus on the first year of study helped to identify the specific challenges that take place during this critical transition period. Specifically, we outline three simultaneous transitions required to succeed in year one of medical school: learning to be a university student, a medical student and a doctor. These transitions were not distinct from each other and overlapped, particularly the medical student and doctor transitions.

Within this common story, a novel six-group typology of students was identified who each struggled with one or more of these transitions. The groups in our typology were: wrong degree choice, mental health problems, acute crisis, at capacity, slow starter and family rock. Many students had both primary and secondary issues. Mental health issues and acute crises were a contributing factor for half of students (*n* = 7).

Our novel typology adds to the literature and existing typologies of strugglers by presenting first-hand evidence of the multi-factorial nature of struggling and how academic issues, personal crises and mental health problems are often inter-linked.

### Comparison with Hays, Lawson and Gray

Overall, our six-group typology maps well to the Hays, Lawson and Gray model [24]. For example, the description of the ‘poor learning skills 1’ student (*school leaver, strong past academic performance, easily coached, variable motivation, challenged by not being in top academic rank at medical school*) is an almost direct match to the at capacity group in our study. The *‘major personal crisis’* group also correlates well to our acute crisis group, and in some cases overlapped with findings from the family rock and mental health problems groups.

The ‘immaturity’, ‘poor organisational skills’ and ‘poor learning skills 2’ profiles all overlap with features of both the at capacity and slow starter groups. For example, Hays, Lawson and Gray delineated a ‘poor organisational skills’ student as exhibiting ‘*poor performance, unable to meet deadlines, insight variable’.*[24] This is comparable to a slow starter in our typology.

Both our typology and the Hays, Lawson and Gray model feature students who struggle with self-regulated learning [24]. Students who do not fully utilise self-regulated learning strategies generally have worse academic outcomes and can become trapped in a cycle of failure due to maladaptive learning habits [35].

Hays, Lawson and Gray separated *‘poor mental health’* into a distinct category. In our study, mental health problems were also a separate group. However, all of the seven students with mental health issues in our study experienced these in combination with other academic or personal problems. This finding may reflect the complex relationship between mental health issues and struggling at medical school, but should be interpreted cautiously given the small numbers of students involved.

The Hays, Lawson and Gray ‘poor insight’ profile does not map to our findings. This profile described a student who has strong self-belief despite poor professional performance, often impervious to feedback and difficult to remediate. Given that the Hays, Lawson and Gray profiles were not specific to first year medical students, it is possible that these behaviours are more likely to emerge later when students spend more time in a clinical environment. An alternative explanation is that students with strong self-belief within the *n* = 52 students who met inclusion criteria may have been unlikely to respond to a study designed to explore experiences of struggling. Therefore, the combination of small sample size and potential for responder bias in our study design may have failed to capture these students.

Hays, Lawson and Gray did not include a group for students who identified that they had chosen the wrong degree programme. These students are a well-established group in other studies of attrition but were a smaller group within our study than in previous research [36].

The family rock group is a new finding when compared to Hays, Lawson and Gray. This finding echoes a previous study of University of Birmingham medical students which highlighted that students with external demands such as family commitments perform less well academically than those that do not, and that this was linked to social affluence. In this previous study, students from the most deprived backgrounds were most likely to have external demands on their time [37].

Overall, our typology derived from first-hand student voices matches up well to a model of strugglers that is almost a decade old. It is particularly comparable on academic factors and acute crises. Given that similar problems are continuing in successive intakes of medical students, we need to reconsider what we know in a new light.

### Professional identity formation

Viewed through the three transitions model presented earlier, students may feel pressured to adopt the ‘doctor’ identity before they have learned how to be a university student or a medical student first. An example of this is students identifying as ‘medics’ from an early stage. In keeping with previous literature, participants reported a clear separation from students on other courses which was both physical and tribal. Gaining admission to this group required agreeing to the prescribed norms [20]. Participants expected an ability to cope with pressure from themselves and other medical students, often articulated via themes of strength and weakness.

Professional identity formation may be happening at an accelerated rate due to the increased and earlier emphasis on professionalism and acquisition of appropriate values and behaviours on medical programmes [21]. From an early stage in the course, students had developed a clear schema in their minds of what a medical student and a doctor could and could not be. A clearly articulated part of this was that for the majority of participants, having mental health issues or seeking help was not compatible with the doctor role.

### Mental health and help-seeking

Reluctance to seek help and specifically the stigma attached to mental health issues corroborate existing literature and are concerning, particularly as they have developed so early [38, 39]. Known barriers which prevent medical students with mental health issues from seeking help include: failure to recognise a problem existed, fear of stigmatization and beliefs about a punitive response from the medical school [38]. All of these themes are repeated in our student narratives.

A reluctance to seek help by some students with significant mental health or personal issues is at odds with the general observation that medical student self-referrals to student support services are increasing [24]. This is complex to unpick as there are tensions between our student voices on this topic and even within individual narratives. Our participants demonstrated a varied pattern of help-seeking behaviours. Some students did not seek help despite encountering significant problems. Of those who sought help, some reported shame or stigma whilst others were more neutral. One student was relieved to access professional help for pre-existing mental health problems for the first time after arriving at university. Signposting to mental health support might be more forthcoming within universities compared to schools due to better awareness amongst staff and students.

### Socio-economic background

In contrast to the dominant narrative of developing a collective professional identity, some study participants described feeling different to their peers. Examples included differences in their family professional background or not attending private education. One participant was admitted via a widening participation route and described feeling unable to reconcile a ‘home life’ with their identity at medical school. This reflects the concept of identity dissonance. Identity dissonance describes students on a professional course who experience internal conflict between their personal identity and the assumption of a professional identity [40]. This can be especially traumatic if one aspect of a student’s identity such as race, gender or socio-economic group is different to the dominant characteristic of the professional body [40, 41]. Our findings regarding identity dissonance speak to existing literature but should be interpreted cautiously as they originated from one participant only.

The importance of family socio-economic circumstances and early academic performance in medicine is increasingly under scrutiny and a substantial literature has reported the challenge facing students from ‘non-traditional’ backgrounds to fit in to elite institutions and professions [42–45]. This student group can be ‘excluded from the inside’ by being admitted to medicine, but not included in it [46]. Students may face inequalities in economic capital, cultural capital (fitting in) and social capital (connections, networks and insider guidance) [47]. These factors are complex to address. Provision of specific mentors from within faculty or other medical students, particularly those from similar backgrounds who have faced similar challenges, can be valued and beneficial for students from this group [48].

## Recommendations for educational practice

### Prospective applicants

Medical school applicants should have accurate expectations of a medical degree programme. A practical way to achieve this would be to pair prospective applicants with a first-year medical student ‘buddy’ over a longitudinal period, for example one year. Buddying schemes often centre on support for the applicant, for example with their personal statement or interview preparation. Ensuring that the focus also included an understanding of the medical student’s day-to-day life, course expectations and assessments would provide a more balanced exchange.

Medical students face specific adaptation challenges including large cohort sizes, a shift away from ‘spoon-fed’ exam-orientated learning to self-directed learning and early professionalism expectations. Medical school communications and marketing such as open days and prospectuses should strive to communicate these realities. Those that support applications including parents, school and sixth form staff should be aware of these issues and how they can contribute to students struggling from an early stage [37].

However, this may be difficult to implement in practice for each of these stakeholders. The university is a business and a place at a specific medical school can be viewed as a product in a competitive marketplace. Universities may be reluctant to communicate any messages that might detract from a course’s appeal. It may also place schools in tension, as many publish the number of university places at elite institutions or on professional programmes such as medicine and law as informal metrics of their performance.

### Early identification and support

Academic staff should strive to identify struggling students at an early stage. Struggling students may be easy to spot if they seek help appropriately or change courses early, but others such as slow starters and those with significant family responsibilities will only be identified via a pro-active approach. The study findings should be shared with academic staff involved in tutoring or pastoral roles, particularly in the first year of medical school. Early identification of these issues could lead to provision of targeted academic and pastoral support, giving each student their best opportunity to succeed on a medical programme.

### Professional identity

The results demonstrate that for many struggling students, knowledge of support services was not an issue. Instead, participants were often unable to overcome barriers to help-seeking. These barriers were rooted in how students perceived the professional identity of a medical student or doctor. Medical school faculty should strive to create an ethos where long-standing pre-conceptions of the doctor’s role are challenged. Changing deeply-ingrained views will be difficult, particularly those that are perpetuated by the hidden curriculum. As a starting point, faculty should engage medical students in active discussion of the above facets of a doctor’s role. This may encourage them to consider an alternative perspective to existing dogma.

### Self-compassion

It is hypothesised that one of the strongest and most consistent positive predictors of successful transition to higher education, particularly for students dealing with mental health problems, is self-compassion [49]. Self-compassion as a concept appears to be at odds with some facets of professional identity articulated by our participants. Moving forwards, self-compassion may be a key area for medical school faculty to target as part of efforts to reframe how professional identity is perceived. This may be a challenging concept to teach didactically- instead it could be introduced through workshops, discussions with tutors or encouraging students to keep a reflective journal.

### Follow up

An additional consideration is the follow up of students who have struggled and gone on to leave the course. There is a well-documented fallout for many students who drop out of medical school [8]. Medical schools have a duty of care to students who struggle and go on to leave the programme [50]. How to implement this practically remains uncertain. One approach could be to have a nominated staff member within a student welfare team to act as a contact point for students who exit the course. This role would require careful consideration of its remit and bespoke training. Students in these circumstances could have a wide set of needs for example help with applying to other university courses or signposting to mental health support. Some exiting students may wish to have no further contact with the medical school and this should be respected. Others may wish to keep in touch and this could be beneficial to both staff and students. Attrition from a medical degree programme is not necessarily a bad outcome if it helps an individual to find a better fit for themselves elsewhere. Profiles of these students and what they go on to achieve after leaving the course may be informative for current students and staff in helping to break down the ‘failed medic’ stereotype.

### Limitations

Data collection took place during one academic year at one UK medical school. Findings are potentially transferrable to similar contexts- specifically, undergraduate medical programmes that admit students directly from secondary school. However, they may not be transferrable to other institutions with different curricula or student demographics. The total number of participants was small (*n* = 14) but this was offset by reaching data saturation [32]. One participant’s data was drawn from an email transcript. This was analysed in the same way as interview transcripts, however these data sources are not directly comparable [51, 52]. The email transcript was short and responses could not be explored further, as they might be in an interview. Email excerpts are highlighted as such in the results and should be interpreted cautiously.

The participant group had a slight over-representation of female students (*n* = 11, 78%) compared to the overall student cohort (approx. 65%). No demographic data was collected for students’ ethnicity and socio-economic group. Study participants were heterogeneous: some chose to leave the course voluntarily and others were asked to leave due to examination failure. This generated a holistic discussion of struggling but may limit the transferability of some findings.

Fourteen of fifty-two students who met inclusion criteria (27%) participated in interviews: responder bias may mean that those who were willing to participate were different to those who were not [53]. The experiences and perspectives of students from this academic year who did not participate in interviews are unknown. This includes students who did not meet inclusion criteria and progressed to the second year of study. Therefore, it is not possible to identify if progressing students experienced similar issues to struggling students and if so how they overcame these.

## Conclusion

This study provides an additional perspective on students who struggle at medical school by focussing on the first year of the course and exploring students’ experience in depth via individual interviews. We have proposed a six-group typology of students who may struggle in their first year of medical school.

Listening to the narratives of students who struggled can help guide the support of future students. Moving forwards, some of the key barriers to overcome relate to how medical students perceive their professional identity. Currently, many students perceive failure, seeking help and having mental health problems as not compatible with working as a doctor. Medical school faculty should strive to challenge these views and put forward an alternative viewpoint. Asking for help when required and learning to cope with failure are not just compatible with working as a doctor: they are essential to safe and successful professional practice.

### Supplementary Information

**Additional file 1.**

**Additional file 2.**

## Data Availability

The dataset generated and analysed during the study contains sensitive content. For this reason and to protect the confidentiality of participants, it has not been made publicly available. The corresponding author can be contacted to discuss potential data access in more detail. Any access requests would require careful consideration. Data sharing could only be carried out if it were to be in accordance with the stipulations outlined in the participants' informed consent process and the study's ethical approval.

## Abbreviations

UK: United Kingdom
MBChB: Bachelor of Medicine and Bachelor of Surgery

## References

[1] James D, Yates J, Ferguson E (2013). Can the 12-item General Health Questionnaire be used to identify medical students who might 'struggle' on the medical course? A prospective study on two cohorts. BMC Med Educ.

[2] Garrud P, Yates J (2012). Profiling strugglers in a graduate-entry medicine course at Nottingham: a retrospective case study. BMC Med Educ.

[3] O’Neill LD, Morcke AM, Eika B (2016). The validity of student tutors’ judgments in early detection of struggling in medical school. A prospective cohort study. Advances in Health Sciences Education.

[4] Li J, Thompson R, Shulruf B (2019). Struggling with strugglers: using data from selection tools for early identification of medical students at risk of failure. BMC Med Educ.

[5] O'Neill LD, Wallstedt B, Eika B, Hartvigsen J (2011). Factors associated with dropout in medical education: a literature review. Med Educ.

[6] Yates J (2012). When did they leave, and why? A retrospective case study of attrition on the Nottingham undergraduate medical course. BMC Med Educ.

[7] Simpson KH, Budd K (1996). Medical school attrition: a 10-year survey in one medical school. Med Educ.

[8] Yates J (2011). Development of a 'toolkit' to identify medical students at risk of failure to thrive on the course: an exploratory retrospective case study. BMC Med Educ.

[9] Sayer M, Chaput De, Saintonge M, Evans D, Wood D (2002). Support for students with academic difficulties. Medical Education.

[10] Lewis AD, Menezes DA, McDermott HE, Hibbert LJ, Brennan SL, Ross EE (2009). A comparison of course-related stressors in undergraduate problem-based learning (PBL) versus non-PBL medical programmes. BMC Med Educ.

[11] Carson AJ, Dias S, Johnston A, McLoughlin MA, O’Connor M, Robinson BL (2000). Mental health in medical students: a case control study using the 60 item General Health Questionnaire. Scott Med J.

[12] Rosal MC, Ockene IS, Ockene JK, Barrett SV, Ma Y, Hebert JR (1997). A longitudinal study of students’ depression at one medical school. Acad Med.

[13] Dyrbye LN, Thomas MR, Shanafelt TD (2006). Systematic Review of Depression, Anxiety and Other Indicators of Psychological Distress Among U.S. and Canadian Medical Students. Academic Medicine.

[14] Dunn LB, Iglewicz A, Moutier C (2008). A conceptual model of medical student well-being: promoting resilience and preventing burnout. Acad Psychiatry.

[15] Tjia J, Givens JL, Shea JA (2005). Factors associated with under-treatment of medical student depression. J Am Coll Heal.

[16] Givens JL, Tija J (2002). Depressed medical students’ use of mental health services and barriers to use. Acad Med.

[17] Guthrie E, Black D, Bagalkote H, Shaw C, Campbell M, Creed F (1998). Psychological stress and burnout in medical students: a five-year prospective longitudinal study. J Roy Soc Med.

[18] Moffat K, McConnachie A, Ross S, Morrison J (2004). First year medical student stress and coping in a problem-based learning medical curriculum. Med Educ.

[19] Ross S, Cleland J, Macleod M (2006). Stress, debt and undergraduate medical student performance. Med Educ.

[20] Cruess RL, Cruess SR, Boudreau JD, Snell L, Steinert Y (2015). A Schematic Representation of the Professional Identity Formation and Socialization of Medical Students and Residents: A Guide for Medical Educators. Acad Med.

[21] Weaver R, Peters K, Koch J, Wilson I (2011). ‘Part of the team’: professional identity and social exclusivity in medical students. Med Educ.

[22] Patel RS, Tarrant C, Bonas S, Shaw RL (2015). Medical students’ personal experience of highstakes failure: case studies using interpretative phenomenological analysis. BMC Med Educ.

[23] Ginsburg S, Regehr G, Lingard L (2003). The Disavowed Curriculum. J Gen Intern Med.

[24] Hays RB, Lawson M, Gray C (2011). Problems presented by medical students seeking support: a possible intervention framework. Med Teach.

[25] Arulampalam W, Naylor RA, Smith JP (2007). Dropping out of medical school in the UK: explaining the changes over ten years. Med Educ.

[26] British Medical Association. Courses at medical school. 2020. https://www.bma.org.uk/advice-and-support/studying-medicine/becoming-a-doctor/courses-at-medical-school. Accessed 15 Feb 2022.

[27] Gale NK, Heath G, Cameron E, Rashid S, Redwood S (2013). Using the framework method for the analysis of qualitative data in multi-disciplinary health research. BMC Med Res Methodol.

[28] Kluge S. Empirically Grounded Construction of Types and Typologies in Qualitative Social Research. Forum Qualitative Sozialforschung / Form: Qualitative Social Research. 2000;1(1). 10.17169/fqs-1.1.1124.

[29] Bryman A. Quantity and Quality in Social Research. London, UK: Unwin Hyman Ltd; 1988. 10.4324/9780203410028.

[30] Addae J, Ettarh R, Crowley J (2020). Study Preferences of Generation Z Students Admitted to Medical School. The Journal of the Federation of American Societies for Experimental Biology.

[31] Lincoln YS, Guba EG (1985). Naturalistic enquiry.

[32] Fusch PI, Ness RL (2015). Are We There Yet? Data Saturation in Qualitative Research. The Qualitative Report.

[33] Cambridge Dictionary. Adulting. 2021. https://dictionary.cambridge.org/dictionary/english/adulting. Accessed 15 Feb 2022.

[34] Cambridge Dictionary. Medic. 2021. https://dictionary.cambridge.org/dictionary/english/medic . Accessed 15 Feb 2022.

[35] Patel R, Tarrant C, Bonas S, Yates J, Sandars J (2015). The struggling student: a thematic analysis from the self-regulated learning perspective. Med Educ.

[36] James D, Chilvers C (2001). Academic and non-academic predictors of success on the Nottingham undergraduate medical course 1970–1995. Med Educ.

[37] Popovic C (2010). Myth busting: an examination of teachers’ beliefs about first-year medical students. How well do teachers know their students?. Innovations in Education and Teaching International.

[38] Winter RI, Patel R, Norman RI (2017). A Qualitative Exploration of the Help-Seeking Behaviours of Students Who Experience Psychological Distress Around Assessment at Medical School. Acad Psychiatry.

[39] Winston KA, Van Der Vleuten CPM, Scherpbier AJJA (2010). At-risk medical students: implications of students’ voice for the theory and practice of remediation. Med Educ.

[40] Costello CY (2004). Changing Clothes: Gender Inequality and Professional Socialization. The National Women's Studies Association Journal.

[41] Monrouxe LV (2010). Identity, identification and medical education: why should we care?. Med Educ.

[42] Reay D (1998). 'Always knowing' and 'never being sure': familial and institutional habituses and higher education choice. J Educ Policy.

[43] Reay D, Davies J, David M, Ball SJ (2001). Choices of Degree or Degrees of Choice? Class, `Race' and the Higher Education Choice Process. Sociology (Oxford).

[44] Mathers J, Parry J (2009). Why are there so few working class applicants to medical schools?. Learning from the success stories.

[45] Greenhalgh T, Seyan K, Boynton P (2004). “Not a university type”: focus group study of social class, ethnic, and sex differences in school pupils' perceptions about medical school. BMJ.

[46] Bourdieu P (1999). The Weight of the World: Social Suffering in Contemporary Society.

[47] Brosnan C, Southgate E, Outram S, Lempp H, Wright S, Saxby T (2016). Experiences of medical students who are first in family to attend university. Med Educ.

[48] Cupitt C, Costello D, Mitchell G. Widening Tertiary Participation Queensland: Student Ambassador Investigations. Perth, WA. National Centre for Student Equity in Higher Education; 2015. https://www.ncsehe.edu.au/publications/widening-tertiary-participation-queensland-student-ambassador-investigations/. Accessed 15 Feb 2022.

[49] Kroshus E, Hawrilenko M, Browning A (2021). Stress, self-compassion, and well-being during the transition to college. Soc Sci Med..

[50] Maher BM, Hynes H, Sweeney C, Khashan AS, O'Rourke M, Doran K (2013). Medical school attrition-beyond the statistics a ten year retrospective study. BMC Med Educ..

[51] Hershberger PE, Kavanaugh K (2017). Comparing Appropriateness and Equivalence of Email Interviews to Phone Interviews in Qualitative Research on Reproductive Decisions. Appl Nurs Res.

[52] Opdenakker R (2006). Advantages and disadvantages of four interview techniques in qualitative research. Forum Qualitative Sozialforschung.

[53] Walsh K (2013). Medical students as human subjects in educational research- the importance of responder bias. Med Educ Online.

