# Correction: Why do students struggle in their first year of medical school? A qualitative study of student voices

**DOI:** 10.1186/s12909-023-04686-3

**Published:** 2023-09-27

**Authors:** Aled Picton, Sheila Greenfeld, Jayne M. Parry

**Affiliations:** Institute of Applied Health Research, College of Medical and Dental Sciences, University of Birmingham, Edgbaston, Birmingham, B15 2TT UK

**Correction: BMC Medical Education (2022) 22:100**

10.1186/s12909-022-03158-4

Following publication of the original article [1], the authors informed us that three issues requiring clarification:

1. In the published paper we have noted that there were ten face-to-face interviews and three telephone interviews. This is incorrect. There were seven face-to-face interviews and six telephone interviews. Our data analysis did not differentiate between data collected in-person or via telephone so we feel that there is no material impact on the data analyses and their interpretation.
2. The ethics statement states that all participants provided written informed consent to participate. This is not fully accurate as those interviewed by telephone provided verbal consent.
3. In the ‘study participants’ section of the results we outlined that other than two participants all ‘had come directly from school to university’. We reached this conclusion based on interview content with each participant e.g. whether or not they made reference to a gap year or previous degree. We concluded that the majority of our participants were school leavers. Although this is likely to be correct it may have been based on assumption and should be interpreted cautiously.
