# Supplementary material for: Why do students struggle in their first year of medical school? A qualitative study of student voices
Source: BMC Med Educ. 2022 Feb 16;22:100. doi: 10.1186/s12909-022-03158-4 (PMC8848907; doi:10.1186/s12909-022-03158-4)
Supplement: Supplementary file 1 — Additional file 1. [file 12909_2022_3158_MOESM1_ESM.docx]

**Appendix 1**

**INTERVIEW PROMPT SHEET**

**MBChB ‘Failure to Proceed’ to Year 2 Study**

**1. INTRODUCTION AND CONSENT**

- Greet participant and introduce self
- Confirm willingness to participate and consent to proceed, including recording of interview
- Remind that participation is voluntary and that they can refuse to answer any questions and/or choose to withdraw at any stage without prejudice
- Complete written consent form
- State that the digital recorder is now switch on

**2. INVITATION TO ‘TELL YOUR STORY’**

The initial part of each interview will be deliberately unstructured in order to allow the interviewee to ‘tell their story’. During this period, JP will note key points (see below) to which they may wish to return later in the interview in order to clarify what has been said or to seek further information. Direct questions (e.g. ‘*did your examination results come as a surprise to you?*) will be avoided until later in the interview and only introduced then if a key point of interest had not been volunteered or addressed. Interviewees will be encouraged to reflect on their responses and to offer their own interpretations of the narratives they tell (e.g. *(When) did you start to find the workload was too much to cope with? What made you think this?)*

The following phrase will be used to open the interview*:*

“Tell me a little bit about your experiences of being a medical student at Birmingham”

*Prompt if interviewee struggles to respond: for example,* “Start wherever you like – why not tell me a bit about why you wanted to become a doctor and why you chose to come to Birmingham?”

If not volunteered, prompt directly on:

- Why medicine as a career?
- Why apply to Birmingham (and where else, and why)?
- Preparedness to come to medical school – how did they do this (website, talk to family, friends, ask other students)?
- Expectations (explore whether any specifics were not anticipated – e.g. peer-examination - or have not materialised – e.g. teaching style, support etc (see later))

**3. KEY EVENTS AND CIRCUMSTANCES**

If not volunteered in response to the opening question, a more direct prompt will be used to begin to surface the factors perceived by the interviewee to have been important in their experiences of Year 1 MBChB; for example:

“What would you say was/were the key things that happened to you last year and which affected the outcome in your final assessments”

*Prompt if interviewee struggles to respond:* for example, “Some students tell us that the things such as family problems at home, financial worries, not enjoying the course or not feeling as though they ‘fitted in’ to the medical school started to affect their ability to study. Did any of these happen to you?”

*Detailed enquiry depending on responses, including for example:*

- “(When) did you begin to think that medicine may not be the right career choice for you? What made you think this?”

*Key factors to explore if not volunteered:* never wanted to do it; not what expected; not ‘fitting in’ with other students. Clarify whether concerns relate to medicine specifically or to university / being away from home more generally

- (When) did you start to find the workload was ‘too much’ to cope with? What made you think this?

*Key factors to explore if not volunteered:* having to work much harder than other students; not enough time to study given other responsibilities at home; needing to hold done a part-time job; living at home so not able to access other students’ support; which particular subjects were difficult; difference in teaching style from previous experiences (e.g. school)

- (When) did you start to think that you didn’t ‘fit in’ with the other students or the medical school? What made you think this?

*Key factors to explore if not volunteered*: perceived social class and pre-University schooling differences; family experience of medicine or university; role models and wider college / university culture

**4. INTERIM ASSESSMENTS AND FEEDBACK**

If not volunteered in response to the opening question, a more direct prompt will be used to explore whether the interviewee were surprised by final assessment outcomes were unexpected; for example:

“Did your examination results come as a surprise to you?

*Prompt if interviewee struggles to respond:* for example, “Some students tell us that they thought that they were doing OK and that they had no idea that they might fail. Was this the way you felt?

**5. HELP AND SUPPORT**

If not volunteered in response to the opening question, a more direct prompt will be used to explore whether the interviewee felt able to identify sources of support and access the necessary help to improve performance; for example:

“Were you able to speak with any one about your concerns about the way your work and exams were going?

*Prompt if interviewee struggles to respond:* for example, “Some students tell us that they weren’t sure who to speak to or whether anyone would be able to help them. Was this the way you felt?

*Detailed enquiry depending on responses, including awareness of, use of and barriers facilitators to use of:*

- Personal Tutor
- Welfare Tutors
- University Student Support
- Other support inside and outside of University

**6. EXTENUATING CIRCUMSTANCES**

We are aware that a number of the students (~n=17) who we wish to invite to participate in the study are presently appealing the Examinations Board decisions on the grounds that there were Extenuating Circumstances which were not made known at the time of the assessment. We understand that the Medical School is supporting most of these appeals. In this part of the interview, we wish to gain insight as to why students did not draw attention to the Extenuating Circumstances now forming the basis of their Appeals.

We will probe with a direct question as to whether or not the interviewee lodged an appeal, and then follow-up with secondary questions depending on their response; for example

“Did you consider appealing the decision of the Examinations Board?”

*(If ‘Yes’)*

“Can you tell me a little bit about the grounds for your appeal?”

“Had you considered telling anyone about [*the extenuating circumstance*] at the time of the exams?”

If an appeal has been lodged, the interviewee will be asked whether they might allow JP to have access to the Appeal documents; for example:

“You will have put a lot of detail in writing in your Appeal documents. It would be helpful for me to be able to look at these documents so as to link up these details with what we’re discussing now. Would you be happy to give me a copy or for me to ask the University for a copy of these documents for use in the Study”

If the interviewee says ‘Yes’

“Thank you. I will send you an email setting it out my request in writing. If you change your mind, that’s absolutely fine – just let me know”.

If the interviewee says ‘No’

“That’s fine; I hope you didn’t mind me asking. Are you OK to continue with the interview?”

**7. OTHER IMPORTANT POINTS**

As the interview moves to conclusion, the interviewee will be given the opportunity to bring anything not already discussed to the table and also to indicate what, it anything, they would do differently; for example:

“Are there any issues that we haven’t discussed that you feel are important for the Study to know about?”

“Is there anything you’d like to feedback to the Medical School which might help future students?”

“What advice would you give to any students coming to read medicine at Birmingham?” Here, prompt on

Whether there is different advice for different periods of time; e.g would help them (a) before they come up to university for the first time in September; (b) in the first few weeks; and (c) before the major assessment period at the end of May/June.

**8. CONCLUSION**

- Thank the interviewee
- Switch off the digital recorder
- Re-iterate the process of withdrawal post-interview
- Ensure has JP’s contact details (on Participant Information Sheet)
