# Supplementary material for: Why do students struggle in their first year of medical school? A qualitative study of student voices
Source: BMC Med Educ. 2022 Feb 16;22:100. doi: 10.1186/s12909-022-03158-4 (PMC8848907; doi:10.1186/s12909-022-03158-4)
Supplement: Supplementary file 2 — Additional file 2. [file 12909_2022_3158_MOESM2_ESM.docx]

| **Appendix 2: Coding framework (V3, final)** | | |
| --- | --- | --- |
| **1 Motivations for applying** |  | **6 Family** |
| 1.1 Open day or campus |  | 6.1 Medicine in family |
| 1.2 Work experience |  | 6.2 University in family |
| 1.3 'Didn't know what else to do' |  | 6.3 Going home in term time |
|  |  | 6.4 Family's perspective on failure/resitting |
| **2 Transition** |  | 6.5 Family's perspective on mental health |
| 2.1 Leaving home |  | 6.6 Letting family down |
| 2.2 Social integration |  | 6.7 Bereavement |
| 2.3 Sixth form versus medical school |  | 6.8 Caring for others not self |
| 2.4 WP* students- joining medical school |  | 6.9 Health problems in family |
| 2.5 WP students- two lives at medical school |  |  |
| 2.6 Graduate entry |  | **7 Health** |
| 2.7 Preparedness |  | 7.1 Impact of physical health problems |
|  |  | 7.2 Mental health- not coping or weakness |
| **3 Studying** |  | 7.3 Medical students shouldn't have problems |
| 3.1 Long contact hours |  | 7.4 Impact of mental health problems |
| 3.2 Isolation of medical school |  | 7.5 Relationship difficulties |
| 3.3 Workload and volume |  | 7.6 Alcohol |
| 3.4 Difficult content |  |  |
| 3.5 Structure and routine |  | **8 Seeking help** |
| 3.6 Staying on top, falling behind |  | 8.1 Asking questions |
| 3.7 Self-directed learning: 'how much?' |  | 8.2 Realisation of own struggle |
| 3.8 Lone working |  | 8.3 Personal mentors |
| 3.9 Collaborative working |  | 8.4 'I don't need help' |
| 3.10 Community based medicine |  | 8.5 'These problems will never happen to me' |
| 3.11 Time management |  | 8.6 Relationships with teaching staff |
| 3.12 What do I need to know? |  | 8.7 EC* application |
| 3.13 Feedback after exams |  | 8.8 Isolation due to problems |
| 3.14 Course etiquette |  |  |
| 3.15 Strategy and gaming |  | **9 Resitting** |
| 3.16 Myths and hidden curriculum |  | 9.1 Adjustment |
| 3.17 Specific learning needs e.g. dyslexia |  | 9.2 Enjoyment |
|  |  | 9.3 Embarassment and shame |
| **4 Year group** |  | 9.4 Separation from original year group |
| 4.1 Size |  | 9.5 Integration into new year group |
| 4.2 Intelligence of other students |  | 9.6 Workload |
| 4.3 Competitive culture |  | 9.7 Confidence and strength |
| 4.4 Perceptions of 'medic culture' |  | 9.8 Other resitting students |
| 4.5 Subgroups |  | 9.9 Struggler to expert |
|  |  |  |
| **5 Living arrangements** |  | **10 Leaving medical school** |
| 5.1 With other medical students |  | 10.1 Never really wanted to do medicine |
| 5.2 With students on other courses |  | 10.2 Don't know what to do |
| 5.3 Ability to study in accommodation |  | 10.3 Changing courses |
| 5.4 Stress in accommodation |  |  |
|  |  | **11 Failure** |
| **WP= widening participation* |  | 11.1 Immediate reaction |
| **EC= extenuating circumstances* |  | 11.2 Never failed anything before |
